# Developing a measure of muscular power during a functional task for older adults

**DOI:** 10.1186/1471-2318-14-145

**Published:** 2014-12-30

**Authors:** Michelle Gray, Sally Paulson

**Affiliations:** Department of Health, Human Performance, and Recreation, Human Performance Laboratory, Office for Studies on Aging, University of Arkansas, HPER 309, Fayetteville, AR 72701 USA; Department of Exercise Science, Shippensburg University of PA, Shippensburg, PA 17257 USA

**Keywords:** Sit-to-stand, Functional movements, Elderly

## Abstract

**Background:**

Muscular power is an important aspect of many activities of daily living and declines at a faster rate than other fitness parameters (i.e. muscular strength and endurance). Assessing muscular power among older adults is problematic as many of the popular tests are contraindicated among older adults and field tests to assess muscular power among older adults have not been validated among older adults. Therefore, the aim of the present investigation was to determine the validity and reliability of a field test to measure of muscular power during a functional movement among community-dwelling older adults (≥ 65 years).

**Methods:**

Twenty community-dwelling older adults (71.6 ± 5.6) volunteered to have their muscular power assessed during repeated sit-to-stand (STS) tasks. Each participant performed 10 STS with 60 s rest between trials. Muscular power was assessed during this functional movement with the Tendo as well as change in center of mass (COM) over time using cinematography.

**Results:**

Relative power measured by Tendo was 5.34 ± 1.67 W/kg and values for COM were 5.39 ± 1.73 W/kg (*p* = .86). Cronbach’s alpha for Tendo muscular power for repeated trials was .98.

**Conclusions:**

Tendo is a simple field method of determining muscular power among older adults and validation is essential. Results from this investigation support Tendo as a valid and reliable method for determining muscular power during a STS task among older adults. Clinicians may use this tool to evaluate and assess progress in older adults’ power and physical functioning.

## Background

By 2030, 71 million Americans will be over 65 years, accounting for approximately 20% of the U.S. population [1]. While the life expectancy of older adults is increasing, the number of healthful years is not. Throughout the aging process, individuals develop functional limitations and physical ability begins to diminish. These declines have been documented for balance [2], gait [3], muscular power [4], as well as cognition [5]. Currently, 80% of older adults are living with at least one mobility disability, and as a result, the cost of health care is three to five times greater than the cost for an individual younger than 65 [1]. While these changes have been attributed to aging, researchers have credited much of these functional declines to reductions in physical activity.

Decreased physical activity has been linked to the development of chronic conditions such as diabetes and high blood pressure as well as increased risk of falls [6]. Morbidity and mortality rates from falls among older adults have risen exponentially over the past decade [7]. In 2009, over 20,000 older adults died from unintentional fall-related injuries [8]. Many of these falls could have been prevented using various exercise training strategies.

Resistance training has been suggested to help increase levels of independent living among older adults [9]. As early as 1998, the American College of Sports Medicine (ACSM) has advocated resistance training for older adults [10] and developed their position stand on muscular power recommendations in 2002 [11]. Both muscular strength and power decline with age [12]. When compared to measures of strength, power declines at a more exponential rate (1-2% vs 3.5% per year, respectively) [13] and is a stronger predictor of mobility disability [14]. Therefore, maintenance of muscular power is imperative for older adults.

While some of these declines in functional ability have been attenuated with exercise, they cannot be prevented completely [15]. However, Bean, Vora, and Frontera [16] provide strong evidence for the implementation of strength and power-based training in older adults to maintain functional independence throughout life. Power training has been shown to increase functional ability more than strength training alone. Specifically, leg power has been reported as a stronger predictor of current functional ability when compared to self-reported physical status of older adults [17]. Not only is current power indicative of current functional status, but loss of muscular power over time is a key predictor for future mobility disability, increased fall risk [18], and decreased independent living [19]. These findings support the concept of evaluating and treating older adults with reduced muscular power as a means to maintain physical functioning.

Power is defined as the product of force and distance divided by the change in time (Force*Distance/Δ Time). In the literature, power is described as a function of strength and speed exhibited by producing elevated forces at rapid speeds [20]. Power has a clear relationship in older adults in reference to their ability to perform activities of daily living (ADLs). Power-based training interventions have been suggested to help older adults maintain functional, independent living, and improve quality of life [21–24].

Valid field tests to determine power and accurately quantify an older adult’s physical ability do not currently exist. Previously, power has been assessed using measures such as vertical jump analysis [25], dynamometers [26], variations of the long jump [27, 28], stair climb tests [29, 30], and the Wingate Cycle test [31, 32]. However, many of these measures are contraindicated for use with many older adults [33]. For younger adults, it has been suggested that assessments of muscular power be performed as specific to the skill being tested as possible. However, this is an issue when assessing older adults, as many of these assessments do not mimic functional activities. Power has been specifically measured in older adults using force plate analysis coupled with cinematography calculating changes in center of mass (COM) during functional tests such as the sit-to-stand (STS) test [34], but these methodologies are not ideal for field-based situations.

The Tendo Weightlifting Analyzer (Trencin, Slovak Republic) has been utilized as a measure of muscular power among younger athletic populations [35], but to our knowledge there have been no data on its ability to measure functional power in older adults. Therefore, the aim of this study was to validate the use of the Tendo Weightlifting Analyzer (Tendo) as a measure of muscular power in a group of community-dwelling older adults.

## Methods

### Subjects

The present investigation employed a cross-sectional design to validate the Tendo Weight Lifting Analyzer as a method of determining muscular power during a functional task among older adults. The target population included community-dwelling older adults (> 65 yrs). Twenty older adults volunteered for the present investigation (Table 1). Subjects were recruited from the community via posted flyers at local community centers, email notification, and word of mouth. All were free from uncontrolled cardiovascular, metabolic, and pulmonary disease. The average age of the sample was 71.6 ± 5.6 years and 60% of the total sample was female. The study was approved by the Institutional Review Board at the University of Arkansas and subjects gave written informed consent prior to their participation.

**Table 1:** **Demographic characteristics of older adults**

Variable	Women (***n*** = 12)	Men (***n*** = 8)	***t***	***p***
Age	69.25 ± 3.08	75.13 ± 6.79	2.642	.02
Weight (kg)	64.69 ± 11.43	87.60 ± 6.10	5.168	.00
Height (cm)	61.67 ± 5.00	69.28 ± 1.58	4.135	.00
Body fat (%)	38.44 ± 6.95	31.70 ± 5.78	-2.266	.04
Tendo (W/kg)	8.28 ± 2.10	7.72 ± 2.59	-0.534	.60
COM (W/kg)	6.11 ± 0.97	7.29 ± 1.64	2.038	.06

Note. Tendo = power (W) relative to body weight (kg) as measured by Tendo Weightlifting Analyzer; COM = center of mass. Results are reported as means ± SD. α = .05.

### Procedures

Upon arrival to the laboratory all subjects completed the Mini Mental State Examination (MMSE) [36] before any other assessments were performed. A cut-off value of 26 was used for the current investigation; no participant scored less than this value. A health history questionnaire was completed and assessed before any physical measures were obtained. Initially, height, weight, and body composition (lean-tissue mass and fat mass) via dual energy x-ray absorptiometry (DXA; GE, Madison, WI, USA) were measured.

### Muscular power

Power was calculated using two different methods (field vs. laboratory) during a STS task that was performed as quickly as possible. During each STS trial, the participant sat on a chair of standard height (0.47 m) with their arms crossed over their chest. Participants were asked to perform 10 STS trials rising from a seated to a full standing position as quickly as possible. Between trials a 60 s rest was performed.

For each of the 10 trials, the Tendo was positioned on the left side of the subject with the Kevlar string in the sagittal plane when the subject was standing. The Tendo was attached to the participant by securing a belt around the subject’s waist. As the subject stood as quickly as possible, the Kevlar string of the Tendo was pulled and average power output (W) for each stand was recorded. From the Tendo, power was assessed from the vertical velocity (m/s) and the mass moved (kg) throughout the complete STS task. For all analyses, relative power was calculated and used for comparisons (W/kg). Each of the 10 trials were recorded and used to calculate reliability. The average of trials 8, 9, and 10 were used to compare the power calculated from the COM values. These three trials were chosen to allow the subjects to become accustomed to the movement. In previous studies (unpublished data), there is a significant difference between the first three trials and last three trials, with those at the end being higher.

To measure power using COM, the subjects were filmed (60 Hz) from the sagittal plane using a 2D motion analysis capture system (Vicon Peak Motus, v. 9.0, Centennial, CO, USA). The digital video camera (Bosch, Dinion, Farmington Hills, MI, USA) was attached to a stationary tripod (height = 0.62 m) positioned perpendicular 3.38 m from the field of view. Prior to each data collection session, a calibration frame was captured and digitized (Peak Motus). A 2D model was created by adhering reflective markers along the right side of the body on the following anatomical locations: acromion process, greater trochanter, lateral joint line of the knee, base of the fifth metatarsal (outside of the shoe), posterior aspect of the heel (outside of the shoe), and lateral malleolus. COM was derived using standard sex body segment length and mass variables. Power was calculated using the subject’s weight (N) expressed as a force, resultant displacement (m) of the COM from the seated to the full standing position, and the time (s) to stand. Time was calculated from the point of initial vertical movement of COM to the highest vertical displacement of COM. Power was calculated using the following equation: (N × m)/s. For all analyses relative power was used (W/kg). Previously, intra-class correlations for muscular power as measured by COM have been reported to be 0.95 [37].

### Statistical analysis

All statistical analyses were conducted using SPSS v. 19.0 (Chicago, IL, USA). Values are reported as mean ± standard deviation. An independent samples t-test was used to detect sex differences between all variables (Table 1). No sex differences existed for either measure of relative power; therefore, all values were grouped together. For power measures, values are reported relative to body weight (W/kg). A dependent t-test was used to assess differences in power between the two methods. Associations between power measured via COM motion analysis and Tendo were determined by using Pearson correlation. Coefficient of reliability was determined for the Tendo Weightlifting Analyzer by using Cronbach’s alpha for the 10 trials performed. Statistical significance was α = .05 for all analyses.

## Results

**Figure 1:**
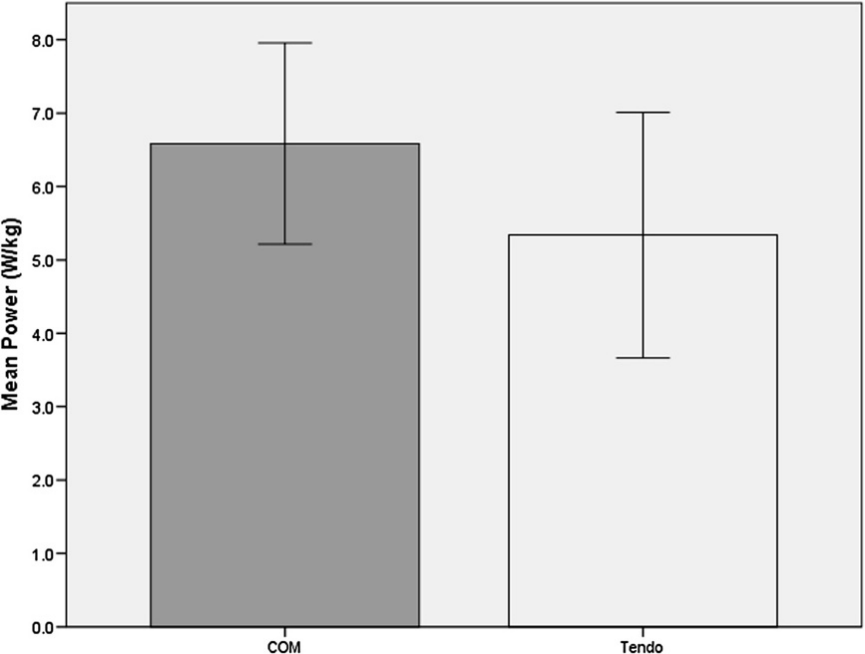
**Differences of mean scores for relative power for each condition.** COM = center of mass as determined by cinematography. Values are reported as means ± SD. α = .05.

**Figure 2:**
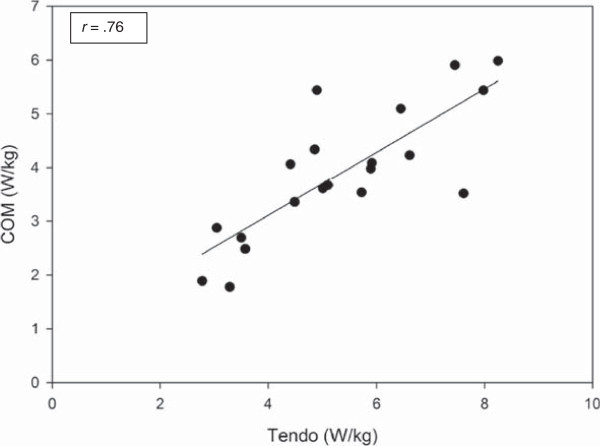
**Scatterplot of and correlation of relative power between conditions.** COM = center of mass as determined by cinematography.

The results of the paired samples t-test indicated relative power was similar for the two methods (*t* = -0.182, *p* = .86; Figure 1). Mean relative power, when calculated with COM and Tendo, was 5.39 W/kg and 5.34 W/kg, respectively. The Tendo Weightlifting Analyzer had average values 0.90% lower when compared to relative power calculated using COM. Measures of STS power were strongly correlated (*r* = .76, *p* < .001; Figure 2).

When assessed for reliability, the Tendo demonstrated a high consistency as a measure for power. The Cronbach’s alpha was .98 for the 10 repeated trials. These results suggest that the Tendo has high reliability between chair stands.

## Discussion

In this sample of community-dwelling older adults, the Tendo muscular power results were similar and strongly correlated to muscular power as measured by COM motion analysis. These results indicate that Tendo is a valid and reliable method of determining muscular power during a STS task among older adults. This information is important to health and fitness professionals that do not have access to COM technology or facilities that need easily accessible measures of muscular power. Test-retest results were high for Tendo, suggesting that it is a reliable measure of muscular power when used in the STS task for older adults.

Recently muscular power among community-dwelling older adults has been reported as low as 1.7 W/kg to as great as 8.64 W/kg when using COM to calculate power [34, 38, 39]. Power in the present investigation was higher than values reported in many previously published articles in community-dwelling older adults. Lindemann and colleagues [34] reported values of 8.64 W/kg when using force plates; but when compared to the power from the Nottingham power rig (4.32 W/kg), results were much closer to the present investigation. The Nottingham power rig has been identified as the ‘gold standard’ assessment of power among older adults; but due to cost it is not available in many laboratories or clinics.

The STS task was chosen for this investigation because of its correlation with functional fitness in older adults [40]. In previous studies, muscular power has been positively correlated with measures of functional fitness [30]. The Nottingham power rig has been reported as the ‘gold standard’ muscular power assessment tool for older adults; however, this method has variable correlation to STS performance [34]. Reported correlations between the power rig and STS performance have ranged from .45 to .83 depending on the population tested [41]. Furthermore, correlations between double leg press power and STS performance have been weak (*r* = .01 - .31) [30, 42, 43]. This is likely because the leg press and the Nottingham power rig assess lower body power in a seated position, which does not take into account potential issues with balance when rising from a seated position. Therefore, results from the power rig may be limited when translating results to functional tasks such as STS performance. The Tendo is a more specific measurement of muscular power as it relates to the STS task since it takes into account deficits in balance that cause an older adult to rise more slowly than when performing a seated power movement.

Similar power results were found in the current investigation when compared to other community-dwelling older adults using a STS task. Power values have ranged from 5.9 W/kg to 8.64 W/kg in other studies [34, 38]. Smith and colleagues [38] reported an average power of 6.4 W/kg during a STS task, compared to 5.36 W/kg in the present investigation. However, they used a force plate to determine average vertical force production to compute muscular power during a STS task compared to body weight (N) in the present investigation. Using maximal ground reaction forces may overestimate muscular power during a STS task.

In the present investigation, the results have limited generalizability. The current sample included only healthy community-dwelling older adults and care should be taken when relating the results to older adults with mobility limitations. In addition, these results were calculated with a small sample.

## Conclusions

Muscular power is an important aspect of functional fitness among older adults. Rarely are activities of daily living performed in a slow, controlled manner; hence, power should be an integral component of any physical assessment performed on an older adult. However, muscular power has been historically a time consuming measure for older adults and the need of sophisticated and expensive equipment has been necessary. While field measures of muscular power, such as vertical jump have been validated and widely used among athletes and young adults, a safe field measure for assessing muscular power among older adults has not previously been devised. Validating the Tendo as an assessment for muscular power is essential for clinicians that may not have access to equipment that is found in many laboratories.

Based on the results, we recommend that Tendo be used to assess muscular power among older adults during the STS task. This task is specifically chosen because of its similarity to many activities of daily living and functional fitness. If an individual is able to perform this task, they are more likely to remain in their homes for a longer period of time.

## References

[1] Jeannotte L, Moore MJ (2007). The State of Aging and Health in America 2007. Centers for Control and Prevention and The Merck Company Foundation.

[2] Balogun JA, Akindele KA, Nihinlola JO, Marzouk DK (1994). Age-related changes in balance performance. Disabil Rehabil.

[3] Oberg T, Karsznia A, Oberg K (1993). Basic gait parameters: reference data for normal subjects, 10–79 years of age. J Rehabil Res.

[4] Galloway MT, Jokl P (1996). Age and sports participation: the effect of aging on muscle function and athletic performance. Sports Med Arthrosc.

[5] Celsis P (2000). Age-related cognitive decline, mild cognitive impairment or preclinical Alzheimer’s disease?. Ann Med.

[6] Nelson ME, Rejeski WJ, Blair SN, Duncan PW, Judge JO, King AC, Macera CA, Castaneda-Sceppa C, American College of Sports M, American Heart A (2007). Physical activity and public health in older adults: recommendation from the American College of Sports Medicine and the American Heart Association. Circulation.

[7] National Institues of Health (2010). Disability in Older Adults: Fact Sheet.

[8] Stevens JA, Corso PS, Finkelstein EA, Miller TR (2006). The costs of fatal and non-fatal falls among older adults. Inj Prev.

[9] Buchner DM, Cress ME, de Lateur BJ, Esselman PC, Margherita AJ, Price R, Wagner EH (1997). The effects of strength and endurance training on gait, balance, fall risk, and health services use in community-living older adults. J Gerontol Med Sci.

[10] Pollock ML, Gaesser GA, Butcher JD, Despres J, Dishman RK, Franklin BA, Garber CE (1998). American college of sports medicine position stand. The recommended quantity and quality of exercise for developing and maintaining cardiorespiratory and muscular fitness, and flexibility in healthy adults. Med Sci Sports Exerc.

[11] Kraemer WJ, Adams K, Cafarelli E, Dudley GA, Dooly C, Feigenbaum MS, Fleck SJ, Franklin B, Fry AC, Hoffman JR, Newton RU, Potteiger J, Stone MH, Ratamess NA, Triplett-McBride T (2002). American college of sports medicine position stand. Progression models in resistance training for healthy adults. Med Sci Sports Exerc.

[12] Seguin R, Nelson ME (2003). The benefits of strength training for older adults. Am J Prev Med.

[13] Skelton DA, Greig CA, Davies JM, Young A (1994). Strength, power and related functional ability of healthy people aged 65–89 years. Age Ageing.

[14] de Vos NJ, Singh NA, Ross DA, Stavrinos TM, Orr R, Fiatarone Singh MA (2005). Optimal load for increasing muscle power during explosive resistance training in older adults. J Gerontol A Biol Sci Med Sci.

[15] Ransdell LB, Vener J, Huberty J (2009). Masters athletes: an analysis of running, swimming, and cycling performance by age and gender. J Exerc Sci Fit.

[16] Bean JF, Herman S, Kiely DK, Frey IC, Leveille SG, Fielding RA, Frontera WA (2004). Increased velocity exercise specific to task (InVEST) training: a pilot study exploring effects on leg power, balance, and mobility in community-dwelling older women. J Am Geriatr Soc.

[17] Foldvari M, Clark M, Laviolette LC, Bernstein MA, Kaliton D, Castaneda C, Pu CT, Hausdorff JM, Fielding RA, Fiatarone-Singh MA (2000). Association of muscle power with functional status in community-dwelling elderly women. J Gerontol Med Sci.

[18] Perry MC, Carville SF, Smith IC, Rutherford OM, Newham DJ (2007). Strength, power output and symmetry of leg muscles: effect of age and history of falling. Eur J Appl Physiol.

[19] Rantanen T, Avela J (1997). Leg extension power and walking speed in very old people living independently. J Gerontol A Biol Sci Med Sci.

[20] Kaminsky LA, Bonzheim KA (2006). ACSM’s Resource Manusal for Guidelines for Exercsie Testing and Prescription.

[21] Bean JF, Leveille SG, Kiely DK, Bandinelli S, Guralnik JM, Ferrucci L (2003). A comparison of leg power and leg strength within the InCHIANTI study: which influences mobility more?. J Gerontol Med Sci.

[22] Weiss A, Suzuki T, Bean J, Fielding RA (2000). High intensity strength training improves strength and functional performance after stroke. Am J Phys Med Rehabil.

[23] Sayers SP, Bean J, Cuoco A, LeBrasseur NK, Jette A, Fielding RA (2003). Changes in function and disability after resistance training: does velocity matter?: a pilot study. Am J Phys Med Rehabil.

[24] Suzuki T, Bean JF, Fielding RA (2001). Muscle power of the ankle flexors predicts functional performance in community-dwelling older women. J Am Geriatr Soc.

[25] Darmiento A, Galpin AJ, Brown LE (2012). Vertical jump and power. Strength Cond J.

[26] Hruda KV, Hicks AL, McCartney N (2003). Training for muscle power in older adults: effects on functional abilities. Can J Appl Physiol.

[27] Izquierdo M, Aguado X, Gonzalez R, Lopez JL, Hakkinen K (1999). Maximal and explosive force production capacity and balance performance in men of different ages. Eur J Appl Physiol.

[28] Thomas JR, Nelson JK, Silverman S, Silverman SJ (2010). Research Methods in Physical Activity.

[29] Hetzler RK, Vogelpohl RE, Stickley CD, Kuramoto AN, Delaura MR, Kimura IF (2010). Development of a modified Margaria-Kalamen anaerobic power test for American football athletes. J Strength Cond Res.

[30] Bean JF, Kiely DK, LaRose S, Alian J, Frontera WR (2007). Is stair climb power a clinically relevant meaasure of leg power impairments in at-risk older adults?. Arch Phys Med Rehabil.

[31] Dallmeijer AJ, Scholtes VA, Brehm MA, Becher JG (2013). Test-retest reliability of the 20-sec Wingate test to assess anaerobic power in children with cerebral palsy. Am J Phys Med Rehabil.

[32] Kohler RM, Rundell KW, Evans TM, Levine AM (2010). Peak power during repeated wingate trials: implications for testing. J Strength Cond Res.

[33] Sands WA, McNeal JR, Ochi MT, Urbanek TL, Jemni M, Stone MH (2004). Comparison of the Wingate and Bosco anaerobic tests. J Strength Cond Res.

[34] Lindemann U, Claus H, Stuber M, Augat P, Muche R, Nikolaus T, Becker C (2003). Measuring power during the sit-to-stand transfer. Eur J Appl Physiol.

[35] Stock MS, Beck TW, DeFreitas JM, Dillon MA (2011). Test-retest reliability of barbell velocity during the free-weight bench-press exercise. J Strength Cond Res.

[36] Cockrell JR, Folstein MF, Copeland JRM, Abou-Saleh MT, Blazer DG (2002). Principles and Pracitve of Geriatric Psychiatry. Principles and Practice of Geriatric Psychiatry.

[37] Hanke TA, Pai Y, Rogers MW (1995). Reliability of measurement of body center-of-mass momentum during sit-to-stand in health adults. Phys Ther.

[38] Smith WN, Del Rossi G, Adams JB, Abderlarahman KZ, Asfour SA, Roos BA, Signorile JF (2010). Simple equations to predict concentric lower-body muscle power in odler adults using the 30-second chair-rise test: a pilot study. Clin Interv Aging.

[39] Zijlstra W, Bisseling RW, Schlumbohm S, Baldus H (2010). A body-fixed-sensor-based analysis of power during sit-to-stand movements. Gait Posture.

[40] Guralnik JM, Ferrucci L, Pieper CF, Leveille SG, Markides KS, Ostir GV, Studenski S, Berkman LF, Wallace RB (2000). Lower extremity function and subsequent disability: consistency across studies, predictive models, and value of gait speed along compared with short physical short performance battery. J Gerontol Med Sci.

[41] Bassey EJ, Fiatarone MA, O’Neill EF, Kelly M, Evans WJ, Lipsitz LA (1992). Leg extensor power and functional performance in very old men and women. Clin Sci (Lond).

[42] Bean JF, Herman S, Kiely DK, Callahan D, Mizer K, Frontera WR, Fielding RA (2002). Weighted stair climbing in mobility-limited older people: a pilot study. J Am Geriatr Soc.

[43] Hardy R, Cooper R, Shah I, Harridge S, Guralnik J, Kuh D (2010). Is chair rise performance a useful measure of leg power?. Aging Clin Exp Res.

[CR44] The pre-publication history for this paper can be accessed here:http://www.biomedcentral.com/1471-2318/14/145/prepub

